# Ontogenic Changes and Differential Localization of T-type Ca^2+^ Channel Subunits Cav3.1 and Cav3.2 in Mouse Hippocampus and Cerebellum

**DOI:** 10.3389/fnana.2016.00083

**Published:** 2016-08-26

**Authors:** Carolina Aguado, Sebastián García-Madrona, Mercedes Gil-Minguez, Rafael Luján

**Affiliations:** Synaptic Structure Laboratory, Department Ciencias Médicas, Instituto de Investigación en Discapacidades Neurológicas (IDINE), Facultad de Medicina, Universidad Castilla-La ManchaAlbacete, Spain

**Keywords:** calcium channels, cerebellum, hippocampus, electron microscopy, immunohistochemistry, quantification

## Abstract

T-type calcium (Ca^2+^) channels play a central role in regulating membrane excitability in the brain. Although the contributions of T-type current to neuron output is often proposed to reflect a differential distribution of T-type channel subtypes to somato-dendritic compartments, their precise subcellular distributions in central neurons are not fully determined. Using histoblot and high-resolution immunoelectron microscopic techniques, we have investigated the expression, regional distribution and subcellular localization of T-type Cav3.1 and Cav3.2 channel subunits in the adult brain, as well as the ontogeny of expression during postnatal development. Histoblot analysis showed that Cav3.1 and Cav3.2 proteins were widely expressed in the brain, with mostly non-overlapping patterns. Cav3.1 showed the highest expression level in the molecular layer (ml) of the cerebellum (Cb), and Cav3.2 in the hippocampus (Hp) and the ml of Cb. During development, levels of Cav3.1 and Cav3.2 increased with age, although there were marked region- and developmental stage-specific differences in their expression. At the cellular and subcellular level, immunoelectron microscopy showed that labeling for Cav3.1 was present in somato-dendritic domains of hippocampal interneurons and Purkinje cells (PCs), while Cav3.2 was present in somato-dendritic domains of CA1 pyramidal cells, hippocampal interneurons and PCs. Most of the immunoparticles for Cav3.1 and Cav3.2 were either associated with the plasma membrane or the intracellular membranes, with notable differences depending on the compartment. Thus, Cav3.1 was mainly located in the plasma membrane of interneurons, whereas Cav3.2 was mainly located in the plasma membrane of dendritic spines and had a major intracellular distribution in dendritic shafts. In PCs, Cav3.1 and Cav3.2 showed similar distribution patterns. In addition to its main postsynaptic distribution, Cav3.2 but not Cav3.1 was also detected in axon terminals establishing excitatory synapses. These results shed new light on the subcellular localization of T-type channel subunits and provide evidence for the non-uniform distribution of Cav3.1 and Cav3.2 subunits over the plasma membrane of central neurons, which may account for the functional heterogeneity of T-type mediated current.

## Introduction

Calcium (Ca^2+^) is a key regulator of neurotransmitter release, neuronal excitation and regulation of gene expression in the brain. The primary pathway of extracellular Ca^2+^ entry in neurons is voltage-gated Ca^2+^ (Cav) channels, classified as high voltage- (HVA: Cav1 and Cav2) and low voltage-activated (LVA: Cav3) channels on the basis of their biophysical properties and their activation thresholds (Catterall, 2000). The Cav3 family (T-type Cav channels) typically activate when neurons are depolarized from relatively hyperpolarized resting potentials, and underlies transient currents that provide a critical contribution to spike output in many neuron types (Perez-Reyes, 2003). The dysfunction of T-type channels has been associated with a number of pathological consequences, and they are therefore important targets for therapy (Zamponi et al., 2015; Zamponi, 2016).

At the molecular level, three pore-forming subunits of T-type channels have been cloned: Cav3.1 (α1G), Cav3.2 (α1H) and Cav3.3 (α1I; Perez-Reyes, 2003). These subunits differ in their voltage-dependent and kinetic properties (McRory et al., 2001; Chemin et al., 2002). Functional T-type channels are composed of just those α1 subunits and do not require assembly with ancillary subunits unlike Cav1/Cav2 channels (Perez-Reyes, 2003). However, they can play diverse roles in the neuronal firing properties due to their ability to form signaling complexes with ion channels, G-protein coupled-receptors (GPCRs) and enzymes (reviewed by Chemin et al., 2006; Iftinca and Zamponi, 2009; Turner and Zamponi, 2014).

*In situ* hybridization studies have established that Cav3.1, Cav3.2 and Cav3.3 subunits are differentially and widely expressed in the brain (Craig et al., 1999; Kase et al., 1999; Talley et al., 1999). Electrophysiological and immunohistochemical studies have demonstrated that T-type channels also exhibit a differential distribution over somato-dendritic domains of central neurons (Carter and Sabatini, 2004; Isope and Murphy, 2005; McKay et al., 2006), suggesting that each Cav3 subunit differentially contributes to T-type responses in distinct subcellular compartments. In the hippocampus (Hp), somatic and dendritic activation of T-type channels in CA1 pyramidal cells initiate LVA Ca^2+^ spikes in response to synaptic activation (Magee et al., 1995; Golding et al., 1999), and can be involved in synaptic plasticity (Isomura et al., 2002). In the cerebellum (Cb), T-type currents control interspike and interburst intervals during spontaneous dendritic burst firing in Purkinje cells (PCs; Pouille et al., 2000; Swensen and Bean, 2003; Womack and Khodakhah, 2004). In dopaminergic neurons, dendritic T-channels provide a Ca^2+^ source for Ca^2+^-activated K^+^ -channels (Wolfart and Roeper, 2002).

In addition to postsynaptic expression and role, Cav3 subunits are distributed at presynaptic sites. Entry of Ca^2+^ through T-type channels into presynaptic nerve terminals is thought to be involved in synaptic exocytosis (Huang et al., 2011; Weiss et al., 2012; François et al., 2015). In each case the distribution of T-type channels over the axo-somato-dendritic axis seems to be a primary determinant of LVA responses, but their pattern of expression and subcellular localization is mostly unknown.

In addition to the adult brain, functional T-type currents are expressed in prenatal and postnatal development (Frischknecht and Randall, 1998; Martin-Caraballo and Greer, 2001; Yunker et al., 2003). Given that the temporal and spatial distribution of ion channels has a unique impact on neuronal properties and plays a role in developmental processes (Luján, 2010; Luján and Aguado, 2015), it is critical to know the neuronal populations expressing T-type channel subunits, their ontogeny and their precise subcellular localization in the brain. Therefore, in order to understand how Cav3.1 and Cav3.2 distribution is organized in the mouse brain, we used histoblots and high-resolution immunohistochemical techniques in combination with quantitative approaches, focusing in the Hp and Cb. Our data demonstrate cell-type differences in the pattern of localization of Cav3.1 and Cav3.2 subunits and their non-uniform distribution in the surface of central neurons.

## Materials and Methods

### Tissue Preparation

The OF-1 mice, from the day of birth [postnatal day (P0)] to adulthood (obtained from the Animal House Facility, School of Medicine, University of Castilla-La Mancha), were used in this study for histoblots and pre-embedding immunohistochemical analyses. The care and handling of animals prior to and during the experimental procedures were in accordance with Spanish (RD 1201/2005) and European Union (86/609/EC) regulations, and the protocols were approved by the University’s Animal Care and Use Committee. In addition, in order to characterize the specificity of our antibody anti-Cav3.2 for immunohistochemistry, two wild-type and three Cav3.2-knockout mice (C57BL6/J strain) were used, kindly made available by Dr. Mala Shah.

For histoblotting, the animals used were from different litters and were grouped as follows: P0, P5, P10, P15, P21, P60 and P90 (*n* = 3 per group). The animals were deeply anesthetized by hypothermia (P0–P5) or by intraperitoneal injection of ketamine/xylazine 1:1 (0.1 mL/kg b.w.) and the brains were quickly frozen in liquid nitrogen. For immunohistochemistry at the electron microscopic level, the animals used were P30 (*n* = 3). The animals were anesthetized by intraperitoneal injection of ketamine/xylazine 1:1 (0.1 mL/kg b.w.) and transcardially perfused with ice-cold fixative containing 4% paraformaldehyde, with 0.05% glutaraldehyde and 15% (v/v) saturated picric acid made up in 0.1 M phosphate buffer (PB, pH 7.4). After perfusion, brains were removed and immersed in the same fixative for 2 h or overnight at 4°C. Tissue blocks were washed thoroughly in 0.1 M PB. Coronal 60 μm thick sections were cut on a Vibratome (Leica V1000).

### Antibodies and Chemicals

The following primary antibodies were used: guinea pig anti-Cav3.1 polyclonal (GP-Af320; aa. 2269–2283 of mouse Cav3.1, C-terminal tail, NM_00978; Frontier Institute Co., Japan) and monoclonal anti-Cav3.2 (N55/10; aa. 1019–1293 of human Cav3.2, cytoplasmic loop, O95180; NeuroMab, UC Davis/NIH, CA, USA). The detailed characterization of the antibody targeting the Cav3.1 channel subunit has been described elsewhere (Hildebrand et al., 2009). The characteristics and specificity of the antibody targeting Cav3.2 has been described elsewhere (Huang et al., 2011) and are also provided in this study.

The secondary antibodies used were as follows: alkaline phosphatase (AP)-goat anti-mouse IgG (H+L) and goat anti-guinea pig IgG (H+L; 1:5000; Sigma-Aldrich, St. Louis, MO, USA), as well as goat anti-guinea pig and anti-mouse IgG coupled to 1.4 nm gold (1:100; Nanoprobes Inc., Stony Brook, NY, USA).

### Histoblotting

The regional distribution of T-type channel subunits was analyzed in rodent brains, using an *in situ* blotting technique (histoblot; Tönnes et al., 1999). For this technique, the expression patterns for Cav3.1 and Cav3.2 were determined in mouse brains. Briefly, horizontal cryostat sections (10 μm) from mouse or rat brain were apposed to nitrocellulose membranes moistened with 48 mm Tris-base, 39 mm glycine, 2% (w/v) sodium dodecyl sulfate and 20% (v/v) methanol for 15 min at room temperature (~20°C). After blocking in 5% (w/v) non-fat dry milk in phosphate-buffered saline, nitrocellulose membranes were treated with DNase I (5 U/ mL), washed and incubated in 2% (w/v) sodium dodecyl sulfate and 100 mm β-mercaptoethanol in 100 mm Tris–HCl (pH 7.0) for 60 min at 45°C to remove adhering tissue residues. After extensive washing, the blots were reacted with affinity-purified anti-Cav3.1 and anti-Cav3.2 antibodies (0.5 mg/mL) in blocking solution overnight at 4°C. The bound primary antibodies were detected with AP-conjugated anti- guinea pig or anti-mouse IgG secondary antibodies. A series of primary and secondary antibody dilutions and incubation times were used to optimize the experimental conditions for the linear sensitivity range of the AP reactions. To compare the expression levels of each protein during development, all nitrocellulose membranes were processed in parallel, and the same incubation time for each reagent was used for all antibodies at all ages. We only compared labeling intensities obtained with the same antibody.

To facilitate the identification of brain regions, structures and cell layers, adjacent cryostat sections were stained with cresyl violet at all developmental ages (not shown). Digital images were acquired by scanning the nitrocellulose membranes using a desktop scanner (HP Scanjet 8300). Image analysis and processing were performed using the Adobe Photoshop software (Adobe Systems, San Jose, CA, USA) as described previously (Ferrándiz-Huertas et al., 2012). The same incubation time for each reagent was used for all antibodies. All of the images were processed with the same equipment in the same way to allow comparison of the intensity of grayscale images at different postnatal ages and in different brain regions on different days. The pixel density (arbitrary units) of immunoreactivity was measured using open circular cursors with a diameter of 0.10 mm. The cursors were placed in different brain regions identified based on the adjacent cresyl violet-stained sections. We used background correction to eliminate potential differences in optical densities across different sections in different experiments. The average of eight background determinations carried out near the brain protein-containing areas of the immunostained nitrocellulose membranes was subtracted from the average pixel densities measured within brain regions. Following background corrections, the average pixel density for the whole region from one animal counted as one “*n*”. Under these conditions, labeling performed on different days produced very consistent results. Data were analyzed and plotted using the software analysis (Soft Imaging Systems, Munster, Germany).

### Immunohistochemistry for Light Microscopy

Immunohistochemical reactions at the light microscopic level were carried out using the immunoperoxidase method as described (Luján et al., 1996). Briefly, sections were incubated in 10% normal goat serum (NGS) diluted in 50 mM Tris buffer (pH 7.4) containing 0.9% NaCl (TBS), with 0.2% Triton X-100, for 1 h. Sections were incubated in anti-Cav3.1 antibodies (1 μg/mL) diluted in TBS containing 1% NGS, followed by incubation in biotinylated goat anti-guinea pig IgG (Vector Laboratories, Burlingame, CA, USA) in TBS containing 1% NGS. Sections were then transferred into avidin-biotin-peroxidase complex (ABC kit, Vector Laboratories, Burlingame, CA, USA). Bound peroxidase enzyme activity was revealed using 3, 3-diaminobenzidine tetrahydrochloride (DAB; 0.05% in TB, pH 7.4) as the chromogen and 0.01% H_2_O_2_ as the substrate. Finally, sections were air-dried and coverslipped before observation in a Leica photomicroscope (DM2000) equipped with differential interference contrast optics and a digital imaging camera (Leica DFC 550).

### Immunohistochemistry for Electron Microscopy

Immunohistochemical reactions for electron microscopy were carried out using the pre-embedding immunogold method described previously (Luján et al., 1996). Briefly, free-floating sections were incubated in 10% (v/v) NGS diluted in TBS. Sections were then incubated in anti-Cav3.1 or anti-Cav3.2 antibodies [3–5 μg/mL diluted in TBS containing 1% (v/v) NGS], followed by incubation in goat anti-guinea pig IgG or anti-mouse IgG coupled to 1.4 nm gold (Nanoprobes Inc., Stony Brook, NY, USA), respectively. Sections were postfixed in 1% (v/v) glutaraldehyde and washed in double-distilled water, followed by silver enhancement of the gold particles with an HQ Silver kit (Nanoprobes Inc., Stony Brook, NY, USA). Sections were then treated with osmium tetraoxide (1% in 0.1 M PB), block-stained with uranyl acetate, dehydrated in graded series of ethanol and flat-embedded on glass slides in Durcupan (Fluka) resin. Regions of interest were cut at 70–90 nm on an ultramicrotome (Reichert Ultracut E, Leica, Austria) and collected on the single slot pioloform-coated copper grids. Staining was performed on drops of 1% aqueous uranyl acetate followed by Reynolds’s lead citrate. Ultrastructural analyses were performed in a Jeol-1010 electron microscope.

### Quantification of Cav3.1 and Cav3.2 Channel Immunoreactivities

To establish the relative abundance of Cav3.1 subunit immunoreactivity in different compartments of PCs and Cav3.2 subunit immunoreactivities in different compartments of CA1 pyramidal cells and PCs at P30, we used 60-μm-thick coronal slices processed for pre-embedding immunogold immunohistochemistry. The procedure was similar to that used previously (Luján et al., 1996; Luján and Shigemoto, 2006). Briefly, for each of three adult animals, three samples of tissue were obtained for the preparation of embedding blocks (totalling nine blocks). To minimize false negatives, electron microscopic serial ultrathin sections were cut close to the surface of each block, as immunoreactivity decreased with depth. We estimated the quality of immunolabeling by always selecting areas with optimal gold labeling at approximately the same distance from the cutting surface. Randomly selected areas were then photographed from the selected ultrathin sections and printed with a final magnification of 45,000×. Quantification of immunogold labeling was carried out in reference areas totalling approximately 2500 μm^2^. Quantification of immunolabeling was performed in three different ways:

#### Percentage of Immunoparticles for T-type Channels

To study the frequency of Cav3.1 and Cav3.2 subunits, we counted immunoparticles identified in each reference area and present in different subcellular compartments: dendritic spines, dendritic shafts and axon terminals. The data were expressed as a percentage of immunoparticles in each subcellular compartment, both in the plasma membrane and at intracellular sites.

#### Density Gradient of T-type Channels Along the Neuronal Surface

To establish the density of Cav3.1 along the surface of PCs and Cav3.2 along the surface of CA1 pyramidal cells and PCs, we performed quantification of immunolabeling in 60-μm-thick coronal slices processed for pre-embedding immunogold. Quantitative analysis of immunogold labeling for Cav3.1 and Cav3.2 in PCs was performed on four different compartments: in PC somata in the PC layer and in apical dendrites (AD), oblique dendrites (OD) and spines in the inner 1/3 of the molecular layer (ml). AD were identified based on their large diameter and the absence of dendritic spines, while OD were identified based on their small diameter and the presence of at least one emerging spine from the dendritic shaft. Quantitative analysis of immunogold labeling for Cav3.1 in CA1 pyramidal cells was performed on eight different compartments: OD and spines in the *stratum oriens (so)*, somata of CA1 pyramidal cells, AD in the *stratum radiatum (sr)* and OD and spines in the *sr* and *strata lacunosum-moleculare (slm)*. Immunoparticles identified in the plasma membrane of pyramidal cells were counted and the area of the subcellular compartment containing the immunoparticles was measured (ImageJ). The data, linear density of Cav3.1 or Cav3.2 in each neuronal compartment, were expressed as the number of immunoparticles/μm.

#### Distribution of T-type Channels Relative to Glutamate Release Sites

To determine the relative abundance of Cav3.1 in dendritic spines of PCs and Cav3.2 in dendritic spines of CA1 pyramidal cells and PCs, and their association with excitatory synapses, immunoparticles identified in each reference area and present in dendritic spines were counted. As differences in the distribution of gold particles among samples were not statistically significant (*P* > 0.31, *Kolmogorov–Smirnov non-parametric test*), the data were pooled. We then measured the length of the dendritic spine membrane (both CA1 pyramidal cells and PCs) from the edge of the synaptic junction. The position of the center of each immunoparticle attached to the plasma membrane of the dendritic spine as a function of distance from the edge of the postsynaptic density was measured using a digitizing tablet and appropriate software (ImageJ). Finally, to obtain a normalized value of the relative abundance of Cav3.1 and Cav3.2 along the dendritic spines, the number of gold particles was expressed as relative frequency in bins corresponding to 60-nm membrane segments of spine membrane.

### Controls

To test the method specificity in the procedures for electron microscopy, antiserum against Cav3.2 was tested on hippocampal and cerebellar sections of Cav3.2 knockout mice. To test the method specificity in the procedures for histoblots and for electron microscopy, the primary antibodies were either omitted or replaced with 5% (v/v) normal serum of the species of the primary antibody. Under these conditions, no selective labeling was observed. Labeling patterns were also compared to those obtained by Calbindin (Swant, Marly, Switzerland); only the antibodies against Cav3.1 and Cav3.2 consistently labeled the plasma membrane.

### Data Analysis

Statistical analyses for morphological data were performed using SigmaStat Pro (Jandel Scientific) and data were presented as mean ± SEM. Statistical significance was defined as *P* < 0.05, as determined using analysis of variance (ANOVA; Kruskal-Wallis test) followed by the Dunns *post hoc* test for comparing all pairs of columns. For the electron microscopic data, statistical significance in the distribution of gold particles among samples was assessed with the Kolmogorov–Smirnov non-parametric test.

## Results

### Differential Regional Expression of Cav3.1 and Cav3.2 Subunits in the Adult Brain

To determine the regional expression of T-type channels in the mouse brain, we used Cav3.1 and Cav3.2 subunit-specific antibodies in conventional immunohistoblotting. Two different anti-Cav3.3 antibodies were also tested, but failed to provide an acceptable or specific signal. Histoblot is now considered a reliable way to analyze the brain expression and regional distribution of different proteins without compromising the integrity of antibody-binding sites by tissue fixation, which is required for standard immunohistochemistry (Tönnes et al., 1999; Fernández-Alacid et al., 2011). When preparing tissue for immunohistochemical techniques, fixation introduces covalent modifications, which may alter the antibody-binding sites. In addition, fixation also introduces cross-linking of proteins, which may hinder the access of antibody to epitopes. However, the direct transfer of native proteins from unfixed frozen tissue sections to an immobilizing matrix offers much improved accessibility of the transferred proteins for immunochemical analysis. The histoblot reflects the spatial pattern in which proteins are arranged within a brain section and affords accurate quantitative analyses in different nuclei of the adult and developing brain, making this technique an attractive alternative, preferred over conventional immunohistochemical techniques.

**Figure 1:**
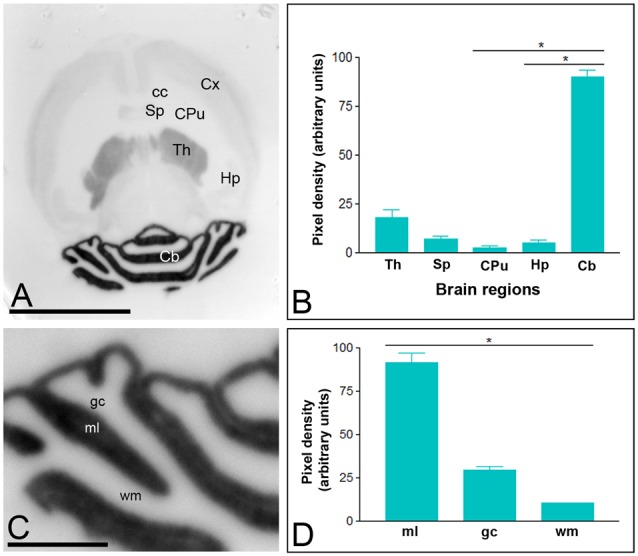
**Regional distribution of the T-type Ca^2+^ channel Cav3.1 subtype in the adult mouse brain**. **(A–D)** The distribution of the Cav3.1 protein was visualized in histoblots of horizontal brain sections at P90 using an affinity-purified anti-Cav3.1 antibody. Cav3.1 expression in different brain regions was determined by densitometric analysis of the scanned histoblots. The strongest expression was detected in the cerebellum (Cb) and thalamus (Th), with weaker expression in the septum (Sp), cortex (Cx) and hippocampus (Hp). The weakest expression level was detected in the caudate putamen (CPu). In the Cb, the strongest expression level was detected in the molecular layer (ml), with lowest intensity in the granule cell layer (gc) and white matter (wm). Error bars indicate SD; *P < 0.05. Scale bars: **(A)**, 0.5 cm; **(C)**, 0.1 cm.

In the adult brain, the overall expression of Cav3.1 and Cav3.2 subunits revealed marked regional-specific differences. Immunoreactivity for Cav3.1 was strongest in the Cb and weaker in the thalamus (Th; Figures 1A,B). Faint staining was observed in the Hp, neocortex and septum (Sp; Figures 1A,B), and very weak labeling in the caudate putamen (CPu; Figures 1A,B). In the Cb, immunoreactivity for Cav3.1 was significantly stronger in the ml than the granule cell layer (gc), in which moderate to weak labeling was consistently detected (Figures 1C,D), and very weak in the white matter (wm; Figures 1C,D).

**Figure 2:**
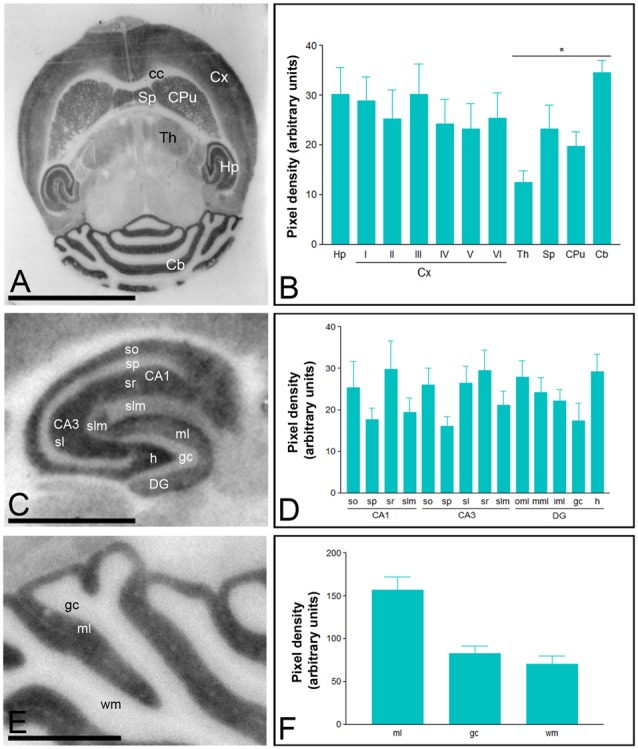
**Regional distribution of the T-type Ca^2+^ channel Cav3.2 subtype in the adult mouse brain.** Protein distribution was visualized on histoblots of brain horizontal sections at P90 using affinity-purified anti-Cav3.2 antibodies. The developed histoblots were scanned and densitometric measurements from five independent experiments were averaged together to compare the protein densities. **(A,B)** The strongest expression was detected in the Cb, Hp, Sp, Cx and CPu, with weaker expression in the Th and no expression in the corpus callosum (cc). In the Cx, the strongest expression levels were found in layer III. **(C,D)** In the Hp, very strong Cav3.2 immunoreactivity was detected in the *strata oriens (so)* and *radiatum (sr)* of the CA1 and CA3 regions, as well as in the hilus and the outer 1/3 of ml of the dentate gyrus (DG). Moderate labeling was observed in the inner 2/3 of ml of the DG, weaker labeling in the *stratum lacunosum-moleculare (slm)* of the CA1 and CA3 regions. The weakest intensity was observed in the *stratum pyramidale* (sp) of the CA1 and CA3 regions and the gc of the DG. In the Cb, the strongest expression level was detected in the ml, and weaker in the gc and wm. Error bars indicate SD; *P < 0.05. h, hilus; sl, *stratum lucidum*. In panels **(E,F)**: gc, granule cell layer; ml, molecular layer; wm, white matter. Scale bars: **(A)**, 0.5 cm; **(C,E)**, 0.1 cm.

Immunoreactivity for Cav3.2 was more widely distributed than Cav3.1 in the brain (Figure 2). Thus, very strong Cav3.2 immunoreactivity was observed in the Hp, Cb, Sp, CPu, and Cx, with moderate labeling in the Th, and weak labeling in the midbrain nuclei, including the inferior and superior colliculi (Figures 2A,B). The neocortex, Hp and Cb, in which the most intense Cav3.2 immunoreactivity was detected, were further examined in region- and layer-specific analyses. In the Cx, the strongest Cav3.2 immunoreactivity was found in layer III, compared to the consistently weaker labeling in layers I, II, IV, V and VI (Figures 2A,B). In the Hp, immunoreactivity for Cav3.2 was very strong in the *so* and *sr* of the CA1 and CA3 and the *stratum lucidum* of CA3 (Figures 2C,D). Moderate labeling was observed in the *slm* of CA3, weak immunoreactivity was evident in the *slm* of CA1 and very weak in the *sp* of the CA1 and CA3 regions (Figures 2C,D). In the dentate gyrus (DG), immunoreactivity for Cav3.2 was very strong in the outer part of the ml, consistently lower in both the medial and inner part of the ml and very weak in the gc (Figures 2C,D). In the Cb, immunoreactivity for Cav3.2 (Figures 2E,F) was significantly stronger in the ml than the gc, and very weak in the wm (Figures 2E,F).

### Expression Patterns of Cav3.1 and Cav3.2 Subunits During Postnatal Development

**Figure 3:**
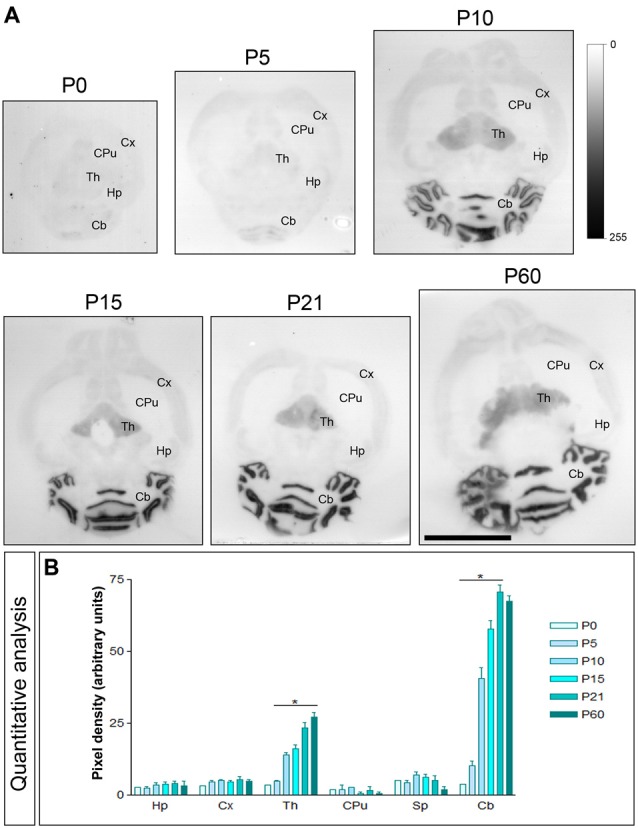
**Developmental and regional distribution of the T-type Ca^2+^ channel Cav3.1 subtype in the mouse brain**. **(A)** Cav3.1 protein distribution was visualized on histoblots of brain horizontal sections at various stages of postnatal development using an affinity-purified anti-Cav3.1 antibody. Cav3.1 was expressed in the brain since the day of birth (P0), and at all stages the strongest expression was detected in the Cb and Th, with the lowest intensity in the CPu. **(B)** The histoblots were scanned and densitometric measurements from four independent experiments were averaged to compare the protein densities for each developmental time point. This quantitative analysis indicates that Cav3.1 expression increased progressively during postnatal development in the Cb and Th. In contrast, Cav3.1 expression does not change significantly from P0 to P60 in the Hp, Cx, CPu and Sp. Error bars indicate SEM; *P < 0.05 compared with P60. Scale bar, 0.5 cm.

The Cav3.1 and Cav3.2 proteins were expressed in the developing brain from the day of birth (P0), showing significant differences in a region- and channel-specific manner. Thus, weak Cav3.1 expression was detected at all developmental stages in the Hp, with only minor changes (Figures 3A,B). Similar expression patterns were detected in other brains regions like cortex (Cx), CPu and Sp (Figures 3A,B). However, in the ml of the Cb, the expression of Cav3.1 increased from its lowest expression at P0 to a peak at P21 and decreased slightly at P60 (Figures 3A,B). A similar developmental expression pattern was found in the Th (Figures 3A,B).

**Figure 4:**
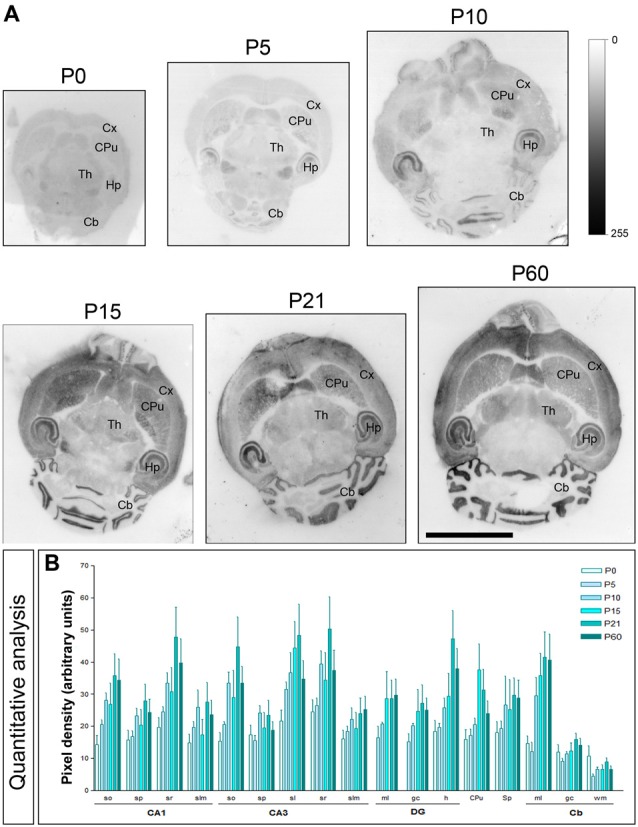
**Developmental and regional distribution of the T-type Ca^2+^ channel Cav3.2 subtype in the mouse brain. (A)** Cav3.2 protein distribution was visualized on histoblots of brain horizontal sections at various stages of postnatal development using an affinity-purified anti-Cav3.2 antibody. Cav3.2 was widely expressed in the brain since the day of birth (P0), and at all stages the strongest expression level was detected in the Hp, Cb and CPu. **(B)** The histoblots were scanned and densitometric measurements from four independent experiments were averaged to compare the protein densities for each developmental time point. This quantitative analysis shows a differential Cav3.2 expression in a developmental stage- and region-specific manner. The CPu and Cb Cav3.2 expression increase with age peaking at P21. In most subfields of the Hp and Sp, the expression increase until P10, decreased at P15 and increased again to peak at P21. so, *stratum oriens*; sp, *stratum pyramidale*; sr, *stratum radiatum*; slm, *stratum lasunosum-moleculare*; sl, *stratum lucidum*; ml, molecular layer; gc; granule cell layer; h, hilus; wm, white matter. CA1, CA1 region of the Hp; CA3, CA3 region of the Hp; DG, dentate gyrus. Error bars indicate SEM. Scale bar, 0.5 cm.

The brain developmental expression of Cav3.2 was different from that described for Cav3.1 above. Thus, in all subfield analyzed in the CA1 region, CA3 region and DG, weak Cav3.2 expression was detected at P0, increased progressively to reach a peak at P21 and then decreased at P60 (Figures 4A,B). At all ages analyzed, labeling for Cav3.2 in dendritic layers was consistently lower in the *slm*, but the weakest subfield was the *sp* in the CA1 and CA3 regions and the gc in the DG (Figures 4A,B). In the ml of the Cb, weak Cav3.2 expression was detected at P0 and P5, increased significantly from P10 to P21, and then decreased to P60 (Figures 4A,B). However, in the cerebellar gc, weak Cav3.2 expression was detected at all developmental stages, with minor changes (Figures 4A,B). Different developmental expression patterns were found in other brain regions. Thus, in the CPu, peak expression for Cav3.2 occurs around P15 as this time point showed maximum expression, while low at other ages (Figures 4A,B). In the Sp, the expression of Cav3.2 increased from its lowest expression at P0 and P5 to reach a plateau from P10 until P60 (Figures 4A,B).

### Subcellular Localization of the Cav3.1 Subunit in the Adult Brain

**Figure 5:**
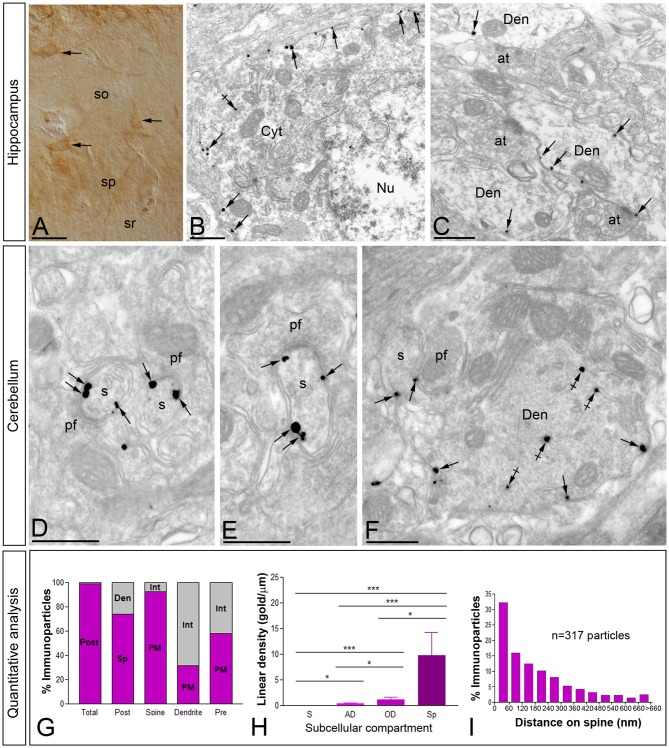
**Cellular and subcellular localization of Cav3.1 in the brain.** Micrographs showing immunolabeling for Cav3.1 in the Hp and the Cb at P30. **(A)** Using the pre-embedding immunoperoxidase at the light microscopic level and differential interference contrast optic, labeling for Cav3.1 was detected in interneurons distributed in the *so* and *sp* of the hippocampal CA1. **(B,C)** In those interneurons, using the pre-embedding immunogold technique at the electron microscopic level, immunoparticles for Cav3.1 were detected along the plasma membrane of somata and dendritic shafts (Den). Few immunoparticles were observed at intracellular sites (crossed arrows). **(D–F)** In the ml of the Cb, immunoparticles for Cav3.1 were abundant on the extrasynaptic plasma membrane (arrows) of dendritic spines (s) of Purkinje cells (PCs), particularly at the edge of postsynaptic densities, contacted by terminals of parallel fibers (pf). In contrast, most immunoparticles for Cav3.1 in dendritic shafts (Den) were detected at intracellular sites (crossed arrows) and few along the extrasynaptic plasma membrane (arrows). **(G–I)** Quantitative analysis showing compartmentalization of Cav3.1 in PCs. **(G)** Bar graphs showing the percentage of immunoparticles for Cav3.1 in PCs. A total of 4306 immunoparticles were analyzed, and virtually all (99%) were postsynaptic. Dendritic spines were enriched in Ca3.1 immunoparticles (74%), of which most were distributed in the plasma membrane (92.3%). In contrast, immunoparticles in dendritic shafts (26%) were mostly distributed at intracellular sites (68.7%). **(H)** Somato-dendritic gradient of Cav3.1 along the surface of PCs. Density of Cav3.1 immunoparticles increased significantly from soma (S) to spines (Sp) of PCs. Error bars indicate SD; *P < 0.05; ***P < 0.001. AD, apical dendrites; OD, oblique dendrites. **(I)** Histogram showing the distribution of immunoreactive Cav3.1 in relation to glutamate release sites in PC dendritic spines. These data show an enrichment of Cav3.1 in the proximity of asymmetrical synapses on dendritic spines. at, axon terminal; Cyt, cytoplasm; Nu, nucleus; sr, stratum radiatum. Scale bars: **(A)**, 50 μm; **(B–F)**, 500 nm.

To establish the precise location of Cav3.1 and Cav3.2 in the brain with high spatial resolution we used pre-embedding immunogold labeling combined with quantitative approaches, focusing on the Hp and Cb. Regarding the cellular localization of Cav3.1 in the Hp, this ion channel was distributed in interneurons located in the *so* and *sp* (Figure 5A), as well as in the *sr*. At the subcellular level, Cav3.1 was localized in the cell bodies and dendritic shafts of interneurons (Figures 5B,C), recognized by the lack of dendritic spines and the convergence of excitatory synapses on the shaft. In those two compartments of interneurons, immunoparticles for Cav3.1 were mainly localized along the somatic plasma membrane and very few at intracellular sites (Figures 5B,C). No significant labeling was detected in presynaptic terminals and axons establishing synaptic contact with interneurons.

In the Cb, the distribution of the Cav3.1 subunit was analyzed in the inner one third of the ml of the cerebellar cortex (Figures 5D–F). Using the pre-embedding immunogold technique, Cav3.1 was primarily detected postsynaptically in dendritic spines associated with the plasma membrane, with very low labeling detected in presynaptic terminals and axons (Figures 5D–F). Quantitative analysis performed on the neuropil showed that from 4.318 immunogold particles analyzed, 4.275 (99%) were distributed at postsynaptic sites and 43 (1%) at presynaptic sites (Figure 5G). Postsynaptically, 61% of all Cav3.1 immunoparticles were in spines and, of those, 92% were located on the plasma membrane and 8% at intracellular sites (Figure 5G). In contrast, in dendritic shafts many immunoparticles were distributed intracellularly associated with membranes (22% of all immunoparticles), small cisterna or vesicles of the endoplasmic reticulum, and a few along the plasma membrane (22% of all immunoparticles; Figures 5F,G).

Next, to quantitatively assess the localization of Cav3.1, PCs were divided into four compartments: soma, AD, OD, and spines. In PCs, the density of Cav3.1 was low in somata (0.03 ± 0.01 immunoparticles/μm), increased 12-folds in AD (0.35 ± 0.1 immunoparticles/μm) and 40-folds in OD (1.18 ± 0.5 immunoparticles/μm; Figure 5H). In dendritic spines of PCs, the density of Cav3.1 was 8-fold higher than that observed on AD (9.74 ± 4.5 immunoparticles/μm; Figure 5H; *p* < 0.001 for soma vs. dendritic spines; *p* < 0.05 for dendritic spines vs. OD; *p* < 0.05 for OD vs. AD, Kruskal–Wallis test and Dunn’s method). Our data shows that the localization of Cav3.1 is not uniform over the dendritic surface of PCs.

Given the high density of Cav3.1 in PC spines, we next analyzed its distribution relative to glutamate release sites (Figure 5I). Immunoparticle counts were then normalized to relative frequency in 60-nm bins. The data showed that about 32% of immunolabeled Cav3.1 is associated with the immediate edge of the synapse, followed by about 47% of all receptors on spines in a 60–300 nm wide band, and then the channel density decreased markedly further in the spine membrane (Figure 5I). This data confirms the association of Cav3.1 with parallel fiber-PC excitatory synapses.

### Subcellular Localization of the Cav3.2 Subunit in the Adult Hippocampus

**Figure 6:**
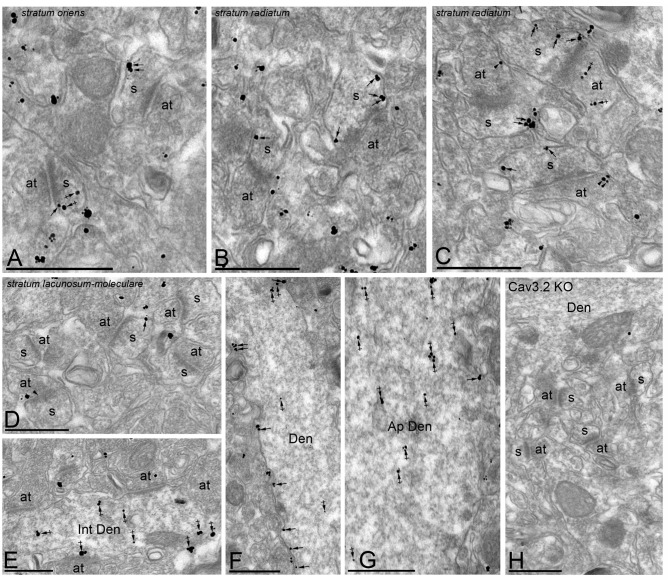
**Cellular and subcellular localization of Cav3.2 in the Hp.** Electron micrographs showing immunoparticles for Cav3.2 in the Hp, as detected using the pre-embedding immunogold technique at P30. **(A–D)** Cav3.2 immunoparticles were abundant on the extrasynaptic plasma membrane (arrows) of dendritic spines (s) of CA1 pyramidal cells contacted by axon terminals (at) in the *so* and *sr* but less frequent in the *slm*. Few immunoparticles were observed at intracellular sites (crossed arrows) in dendritic spines. Immunoparticles for Cav3.2 were also localized to the extrasynaptic plasma membrane (arrowheads) of axon terminals (at) establishing asymmetrical synapses with spines (s). **(E–G)** In dendritic shafts, immunoparticles for Cav3.2 were mainly found in the plasma membrane in oblique dendrites (OD) but mainly detected at intracellular sites (crossed arrows) in apical dendrites (AD). Cav3.2 immunoparticles were also detected in interneurons (Int Den) at intracellular sites (crossed arrows). **(H)** The antibody specificity was controlled and confirmed in the Hp of Cav3.2 KO mice that were free of any immunolabeling, with the exception of very few particles associated to mitochondria (arrow). Scale bars: **(A–G)**, 500 nm.

In the Hp, the localization of the Cav3.2 subunit was analyzed in all dendritic subfields of the CA1 region (Figure 6). Cav3.2 immunoparticles were found along the extrasynaptic plasma membrane of dendritic spines and shafts, and associated with membranes at intracellular sites (Figure 6). Dendritic spines in the *so* and *sr* were strongly labeled for Cav3.2 (Figures 6A–C) and the labeling intensity decreased significantly in the *slm* (Figure 6D). Immunoparticles appeared occasionally at the edge of asymmetrical synapses on dendritic spines (Figures 6A–C). Immunoparticles for Cav3.2 were also found along the extrasynaptic plasma membrane of OD (Figure 6F), but mainly distributed at intracellular sites in AD (Figure 6G). In addition to pyramidal cells, immunoreactivity for Cav3.2 was also detected in dendritic shafts of interneurons, although most immunoparticles were distributed at intracellular sites (small cisterna or vesicles of the endoplasmic reticulum) and very few in the plasma membrane (Figure 6E). Presynaptically, immunoreactivity for Cav3.2 was detected in axon terminals making asymmetrical synapses with dendritic spines throughout all dendritic layers (Figures 6C,D). Specificity of immunolabeling in the Hp using the pre-embedding immunogold technique was confirmed in samples of Cav3.2 KO mice (Figure 6H).

**Figure 7:**
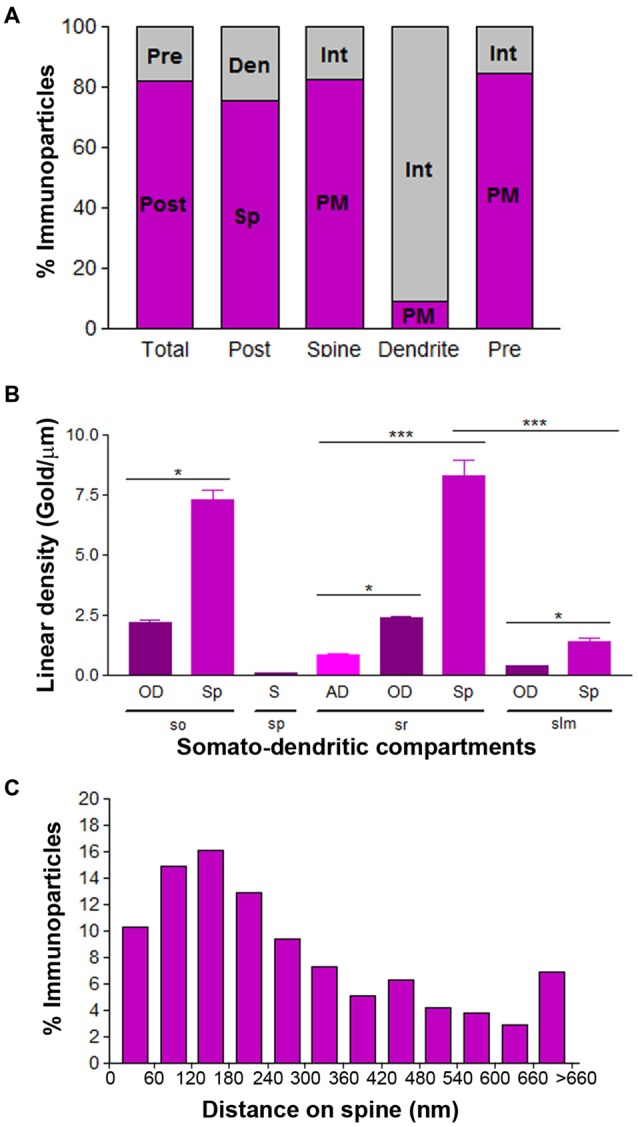
**Compartmentalization of Cav3.2 in CA1 pyramidal cells. (A)** Bar graphs showing the percentage of immunoparticles for Cav3.2 at post- and presynaptic compartments and along plasma membrane vs. intracellular sites. A total of 3086 immunoparticles were analyzed, and 82.2% were postsynaptic and 17.8% presynaptic. Postsynaptically, 75.4% were detected in dendritic spines and 24.6% in dendritic shafts. Most immunoparticles in spines were in the plasma membrane (82.4%) and most in dendritic shafts were at intracellular sites (90.8%). **(B)** Change in the density of Cav3.2 in CA1 pyramidal cells as a function of distance from the soma. Density of Cav3.2 immunoparticles increased significantly from soma (S) to dendritic spines (Sp) in the *so* and *sr*, but not in the *slm*. Error bars indicate SD; *P < 0.05; ***P < 0.001. AD, apical dendrites; OD, oblique dendrites; sp, *stratum pyramidale*. **(C)** Histogram showing the distribution of immunoreactive Cav3.2 in relation to glutamate release sites in dendritic spines of CA1 pyramidal cells. Data are expressed as the proportion of immunoparticles at a given distance from the edge of the synaptic specialization. These data show that 64% of all Cav3.2 immunoparticles were distributed within the first 300 nm from the edge of asymmetrical synapses.

To quantitatively assess the localization of Cav3.2 in CA1 pyramidal cells, we first calculated the proportion of immunoparticles in the plasma membrane and at intracellular sites. From 3.087 immunogold particles analyzed, 2.539 (82%) were distributed at postsynaptic sites and 548 (18%) at presynaptic sites (Figure 7A). Postsynaptically, 75% of all Cav3.2 immunoparticles were in spines and, of those, 82% were located on the plasma membrane and 18% at intracellular sites (Figure 7A). In contrast, in dendritic shafts many immunoparticles were distributed intracellularly associated with membranes (25% of all immunoparticles) and few along the plasma membrane (9% of all immunoparticles; Figure 7A).

Next, we performed a quantitative comparison of the Cav3.2 densities in eight somato-dendritic domains of CA1 pyramidal cells (Figure 7B). Quantification of immunogold reactions demonstrated a distance-dependent increase from soma (0.08 ± 0.03 immunoparticles/μm) to dendritic spines in both the *so* (7.30 ± 1.8 immunoparticles/μm) and *sr* (8.29 ± 3.0 immunoparticles/μm), with a significant decrease in dendrites of the *slm* (1.42 ± 0.7 immunoparticles/μm; Figure 7B; *p* < 0.001 for soma vs. dendritic spines in *so*; *p* < 0.001 for soma vs. dendritic spines in *sr*, Kruskal–Wallis test and Dunn’s method). The densities of immunoparticles in OD and dendritic spines showed a similar distance-dependent increase in the *so* and *sr* (Figure 7B; *p* > 0.05 for OD in *so* vs. OD in *sr*; *p* > 0.05 for dendritic spines in *so* vs. dendritic spines in *sr*, Kruskal–Wallis test and Dunn’s method).

Finally, to analyze the location of Cav3.2 immunoparticles relative to glutamate release sites, the distance from the center of the particles to the edge of excitatory synapses were measured in CA1 dendritic spines (Figure 7C). The data showed that about 64% of immunolabeled Cav3.2 are located in a 60–300 nm wide band, and then the channel density decreased markedly further in the spine membrane (Figure 7C).

### Subcellular Localization of the Cav3.2 Subunit in the Adult Cerebellum

**Figure 8:**
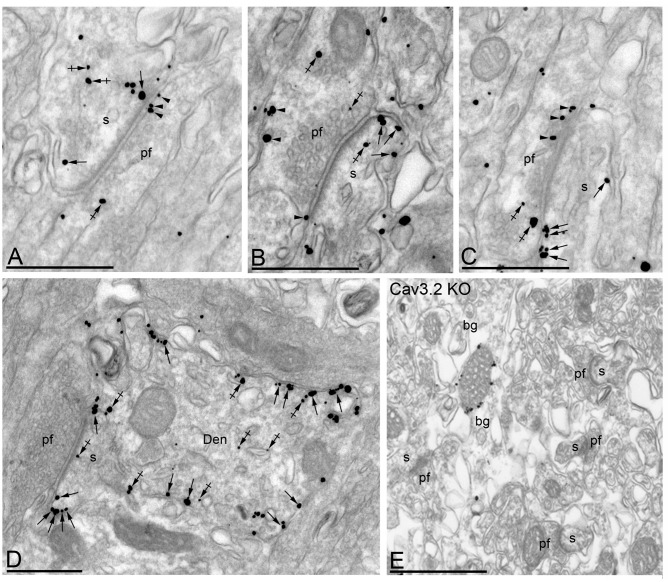
**Cellular and subcellular localization of Cav3.2 in the Cb**. Electron micrographs showing immunoparticles for Cav3.2 in the ml of the Cb, as detected using the pre-embedding immunogold technique at P30. **(A–C)** Immunoparticles for Cav3.2 were mostly distributed along the extrasynaptic plasma membrane (arrows) of dendritic spines (s) of PCs contacted by terminals of parallel fibers (pf), and to a lesser extent at intracellular sites (crossed arrows) in PC spines. Cav3.2 immunoparticles were also distributed presynaptically, in axon terminals of pf. Most of these presynaptic immunoparticles were localized in the plasma membrane (arrowheads) of active zone and extrasynaptically, and a few were distributed at intracellular sites (crossed arrows). **(D)** In dendritic shafts (Den), immunoparticles for Cav3.2 were more frequently detected at intracellular sites (crossed arrows) than along the extrasynaptic plasma membrane (arrows). **(E)** The antibody specificity was controlled and confirmed in the Cb of Cav3.2 KO mice that were free of any immunolabeling, with the exception of very few particles associated to mitochondria (arrow). Scale bars: **(A–E)**, 500 nm.

**Figure 9:**
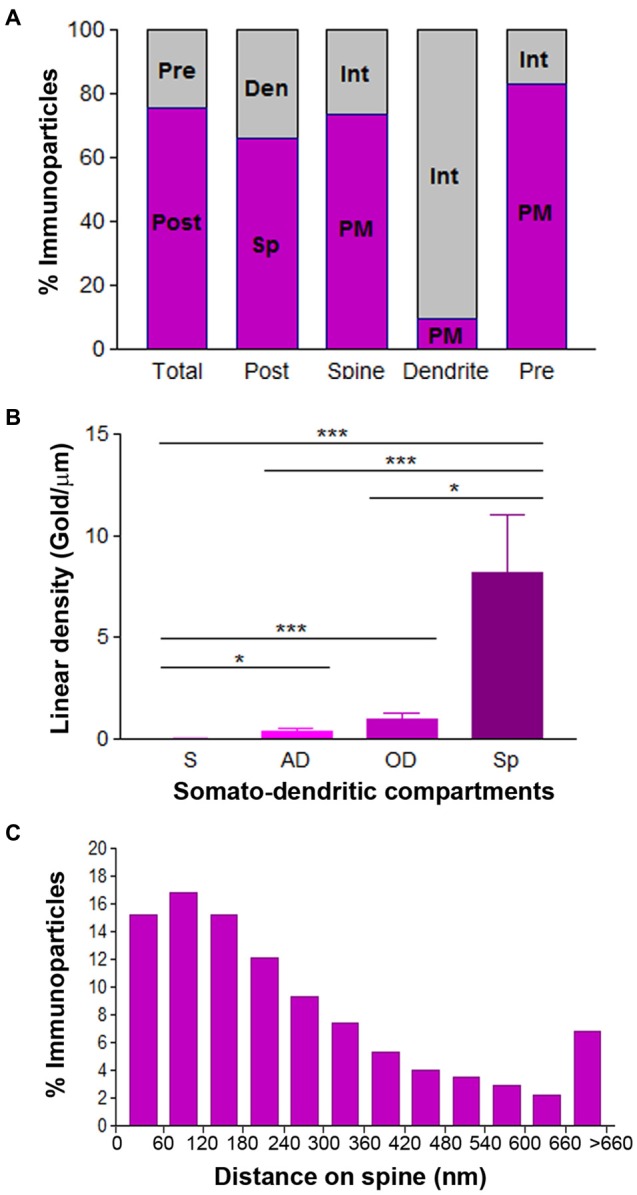
**Compartmentalization of Cav3.2 in PCs**. **(A)** Bar graphs showing the percentage of immunoparticles for Cav3.2 in PCs. A total of 3123 immunoparticles were analyzed, and 75.7% were postsynaptic and 24.3% presynaptic. Postsynaptically, 66.1% were detected in dendritic spines and 33.9% in dendritic shafts. Most immunoparticles in spines were in the plasma membrane (73.3%) and most in dendritic shafts were at intracellular sites (90.5%). **(B)** Change in the density of Cav3.2 in PCs as a function of distance from the soma. Density of Cav3.1 immunoparticles increased significantly from soma (S) to spines (Sp) of PCs. Error bars indicate SD; *P < 0.05; ***P < 0.001. AD, apical dendrites; OD, oblique dendrites. **(C)** Histogram showing the distribution of immunoreactive Cav3.2 in relation to glutamate release sites in PC dendritic spines. These data show that 69% of all Cav3.2 immunoparticles were distributed within the first 300 nm from the edge of asymmetrical synapses.

In the Cb, the distribution of the Cav3.2 subunit was analyzed in the inner one third of the ml of the cerebellar cortex (Figure 8). Cav3.2 was mainly detected postsynaptically along the extrasynaptic plasma membrane of dendritic spines and associated with intracellular membranes (Figures 8A–D). This was confirmed using quantitative analyses, which showed that 66% of all Cav3.2 immunoparticles were in spines and, of those, 74% were located on the plasma membrane and 27% at intracellular sites (Figure 9A). Many immunoparticles were detected close to the edge of asymmetrical synapses on dendritic spines (Figures 8A–D). In dendritic shafts, immunoparticles were frequently observed at intracellular membranes of the endoplasmic reticulum (91% of all immunoparticles) and to a lesser extent in the plasma membrane (9% of all immunoparticles; Figures 8D, 9A). At presynaptic sites, immunoreactivity for Cav3.2 was detected in parallel fiber terminals establishing excitatory synapses with dendritic spines of PCs (Figures 8A–C), and represented 24% of all immunoparticles (Figure 9A). Specificity of immunolabeling in the cerebellar cortex using the pre-embedding immunogold technique was confirmed in samples of Cav3.2 KO mice (Figure 8E). No specific immunolabeling for Cav3.2 was found in PCs, although the same antibody under identical experimental conditions provides non-specific labeling associated with mitochondria in morphologically identified Bergmann glia (Figure 8E).

Given the non-uniform distribution of Cav3.2 in CA1 pyramidal cells, we analyzed whether the ion channel has a similar somato-dendritic gradient in PCs. For this purpose, we determined the density of Cav3.2 immunoparticles in four different compartments of PCs: soma, AD, OD, and spines. Quantification of immunogold reactions demonstrated a distance-dependent increase from soma to dendritic spines (Figure 9B). Thus, dendritic spines (8.16 ± 2.8 immunoparticles/μm) showed 289 times higher density of Cav3.2 immunoparticles than soma (0.03 ± 0.01 immunoparticles/μm), 21 times higher than AD (0.38 ± 0.1 immunoparticles/μm) and 8 times higher than OD (0.97 ± 0.3 immunoparticles/μm; Figure 9B; *p* < 0.001 for soma vs. dendritic spines; *p* < 0.05 for dendritic spines vs. oblique dendrites; *p* < 0.05 for OD vs. AD, Kruskal–Wallis test and Dunn’s method). Finally, the location of Cav3.2 immunoparticles relative to glutamate release sites in PC spines was also analyzed (Figure 9C). Although only 15% of immunoparticles were located adjacent to the PSD within the first 60 nm, a large proportion of them (about 67%) were located within 300 nm (Figure 9C). Then, the channel density decreased markedly further in the spine membrane (Figure 9C).

## Discussion

A major functional distinction between Cav3 channels and Cav1/Cav2 channels is their differing activation threshold. While Cav1/Cav2 channels normally require a highly potent depolarization to be activated, Cav3 channels respond to small changes in membrane potential (Perez-Reyes, 2003). In neurons, Cav3 channel subunits regulate the excitability of the plasma membrane. Using subunit-specific antibodies, our study established the expression and localization of Cav3.1 and Cav3.2 in key brain regions reported to express these Ca^2+^ channels and to generate T-type-mediated responses. Our histoblot study has established the differential expression pattern of Cav3.1 and Cav3.2 subunits in the brain in a region- and developmental stage-manner. The Hp best illustrates this differential expression pattern between Cav3.1 and Cav3.2, but in the cerebellar cortex they show an overlapping pattern. Our immunoelectron microscopy study has revealed the quantitative distribution of the Cav3.1 and Cav3.2 subunits in different subcellular compartments in CA1 pyramidal cells and PCs. The results show that both Cav3.1 and Cav3.2 subunits are distributed with a non-uniform density over the somato-dendritic surface of the CA1 pyramidal cells and PCs. In addition, Cav3.2 but not Cav3.1 is localized at presynaptic sites. Our results are consistent with the notion that differential distribution of T-type channel subunits may account for the functional heterogeneity observed in the brain.

### Differential Expression of Cav3.1 and Cav3.2 Subunits in the Adult and Developing Brain

T-type channels are pharmacologically and physiologically heterogeneous (Zamponi et al., 2015) and this fact lead to the idea that such variability may result, at least in part, from differential expression of individual channel subtypes in distinct nuclei and neuron populations. Our histoblot analysis demonstrated that Cav3.1 and Cav3.2 proteins were widely expressed throughout the brain, consistent with previous *in situ* hybridization (Craig et al., 1999; Talley et al., 1999) and immunohistological studies (Craig et al., 1999; Yunker et al., 2003; McKay et al., 2006). We showed that the Cav3.1 and Cav3.2 proteins were expressed in different brain regions where T-type activity has been demonstrated using electrophysiological techniques. These regions include the Hp, Cb, Cx, CPu, Sp and thalamic nuclei, and in all of them T-type channels are implicated in the generation of low-threshold spikes (Coulter et al., 1989; Magee and Johnston, 1995; Mouginot et al., 1997; Niespodziany et al., 1999; Williams et al., 1999; Zhou and Antic, 2012).

At the protein level, the intensity of immunolabeling for Cav3.1 and Cav3.2 varied markedly between specific brain regions and showed notable regional differences such as in the Hp and CPu. However, the two channel subtypes displayed overlapping expression in other regions, particularly in the Cb and thalamic nuclei. In the Cb, strong immunoreactivity for Cav3.1 and Cav3.2 was observed in the ml, suggesting their expression in PCs. In agreement with our data, previous mRNA and protein studies have shown the expression of the Cav3.1, Cav3.2 and Cav3.3 subunits in PCs (McKay et al., 2006; Molineux et al., 2006). However, some discrepancies were also be detected regarding the mRNA and the protein. Thus, *in situ* hybridization techniques show that the Cav3.1 is highly expressed in pyramidal cells of the CA1 and CA3 regions and granule cells of the DG, as well as granule cells of the cerebellar cortex (Talley et al., 1999, 2000). We observed a very low level of expression in the Hp, and no expression in granule cells of the Cb, in agreement with previous immunohistochemical data using validated antibodies (Hildebrand et al., 2009). Regarding Cav3.2, the mRNA has been described to be expressed at low levels in PCs of the Cb (Talley et al., 1999), while we found a high expression at the protein level.

During development, Ca^2+^ and Cav channels are involved in a number of functions including neurogenesis, neurites outgrowth, synapse formation and elimination, cell differentiation, and neuronal death (reviewed by Rosenberg and Spitzer, 2011; Uhlén et al., 2015). Whereas the developmental expression of Cav1/Cav2 channels is mostly known (Ludwig et al., 1997; Ferrándiz-Huertas et al., 2012), data regarding the temporal expression of T-type channels are very scarce, and this information is critical to understand the molecular basis of Cav signaling during brain development. In our study, expression of Cav3.1 and Cav3.2 was detected in the brain since the day of birth and increased throughout postnatal development, consistent with previous studies using western blots for Cav3.1 in forebrain and hindbrain (Yunker et al., 2003), and showing an increase in T-type-mediated currents in the Hp (Thompson and Wong, 1991; Kortekaas and Wadman, 1997). In addition, the large spatial resolution of the histoblot technique (Fernández-Alacid et al., 2011) allowed us to detect marked differences in the developmental expression profiles in a region- and layer-specific manner. For instance, we observed that the developmental profiles of Cav3.2 were different at specific ages in the Hp, CPu or Sp. To our knowledge, this data provide the first detailed description of the changes in T-type channel expression in the brain during postnatal development.

### Cellular and Subcellular Localization of Cav3.1 and Cav3.2 Subunits in the Brain

The subcellular localization of a given ion channel depends on the cell type and its role within the neuronal circuit (Luján, 2010; Luján and Aguado, 2015). Central neurons express many types of ion channels, whose distribution patterns over the axo-somato-dendritic plasma membrane dramatically affect neuronal function (Luján, 2010; Luján and Aguado, 2015). Among all ion channels, Ca^2+^ channels are of particular interest because their subcellular localization can also impact on the entry of Ca^2+^, which triggers the activation of Ca^2+^-dependent enzymes, the secretion of neurotransmitter or second messenger cascades important for many cellular processes (Perez-Reyes, 2003; Zamponi et al., 2015).

At the cellular level, we detected T-type channels in three main neuron populations: CA1 pyramidal cells and interneurons in the Hp, and PCs in the Cb. Cav3.2 was detected in CA1 pyramidal cells, but both Cav3.1 and Cav3.2 were found in PCs and hippocampal interneurons. Although the Cav3.1 mRNA seems to be expressed in pyramidal cells (Craig et al., 1999; Talley et al., 1999) we did not find detectable levels of the protein using our immunohistochemical techniques, in agreement with the findings described by Hildebrand et al. (2009), as well as Yunker et al. (2003) that used a different antibody anti-Cav3.1. Nevertheless, our cellular expression of T-type channels is consistent with previous immunohistochemical studies (Craig et al., 1999; McKay et al., 2006), although they also differ in some aspects for the Cav3.2 distribution. The former studies, which did not perform any tests in KO mice for the specificity of their antibodies, showed a strong somata and dendritic labeling in rats, while using specific antibodies we found low labeling in somata but strong in the neuropil in mice. This particular neuropilar labeling pattern has been demonstrated for other ion channels in the brain (see for instance Fernández-Alacid et al., 2011; Ballesteros-Merino et al., 2012, 2014; Ferrándiz-Huertas et al., 2012) by many laboratories. Other than the antibody specificity, the reason for this discrepancy might reflect differences in the species used or in the immunohistochemical techniques employed. Finally, we provide ultrastructural evidence for the presence of both Cav3.1 and Cav3.2 in hippocampal interneurons, but with two very different distribution patterns. While Cav3.1 was mainly located in the plasma membrane, the plausible location where channels could respond to synaptic depolarization to mediate Ca^2+^ currents, Cav3.2 was mainly located at intracellular sites. In agreement with our data, previous studies indicated that interneurons can generate T-type-mediated current (Lacaille and Williams, 1990; Fraser and MacVicar, 1991), and morphological studies showed that this function can be performed by Cav3.1 channels (Yunker et al., 2003; Vinet and Sík, 2006).

At the subcellular level, in accordance with the histoblot analyses showing a similar expression pattern of Cav3.1 and Cav3.2 in the cerebellar cortex, immunoelectron microscopy revealed a comparable subcellular distribution of these two subunits in postsynaptic compartments of PCs. Thus, both Cav3.1 and Cav3.2 were more frequently observed in the plasma membrane of dendritic spines and at intracellular sites in dendritic shafts, as also happened in CA1 pyramidal cells for Cav3.2. These findings are consistent with previous studies showing Cav3.1 and Cav3.2 in somato-dendritic domains of the two neuron populations (Craig et al., 1999; McKay et al., 2006; Molineux et al., 2006), as well as in neurons of the spinal cord (François et al., 2015). On the other hand, our immunoelectron microscopic study showed that most of the labeling for Cav3.1 and/or Cav3.2 in dendritic shafts of CA1 pyramidal cells, interneurons and PCs were found intracellularly associated with intracellular membranes. The large amount of intracellular labeling could represent newly synthesized Cav3.2 translocating from the site of synthesis to the plasma membrane or channels undergoing internalization from the site of action.

In addition to the well characterized presence of T-type currents in somato-dendritic compartments of neurons, we also found presynaptic labeling for Cav3.2 but not Cav3.1 channels in axon terminals establishing excitatory synapses in the Hp and Cb. Other studies also failed to detect Cav3.1 at presynaptic sites in the Cb and Th (Hildebrand et al., 2009; Parajuli et al., 2010). Presynaptic labeling for Cav3.2 channels has been shown in other brain regions, including the Hp (Tang et al., 2011), entorhinal Cx (Huang et al., 2011), spinal cord (Jacus et al., 2012; François et al., 2015) and peripheral nerve endings (Rose et al., 2013). Presynaptic activation of T-type channels is thought to result primarily in the control of spontaneous vesicular release (Huang et al., 2011; Tang et al., 2011; Jacus et al., 2012; Rose et al., 2013). Altogether, our data suggest that presynaptic effects of T-type channels might be molecularly mediated by the Cav3.2 subunit.

### Non-Uniform Distribution of Cav3.1 and Cav3.2 Subunits in Neurons

Quantitative comparison of immunogold densities showed that Cav3.1 and Cav3.2 followed a similar somato-dendritic gradient in the PCs. The density of Cav3.1 and Cav3.2 immunoparticles increased significantly from the soma towards dendritic spines, suggesting that PC uses similar mechanisms to target and traffic the two T-type channels. The same somato-dendritic gradient was found for Cav3.2 in CA1 pyramidal cells. However, in this neuron type, the distribution did not follow a proximal-to-distal gradient, as the density of Cav3.2 decreased significantly in the distal dendrites of pyramidal cells located in the *slm*. The non-uniform distribution of Cav3.1 and Cav3.2 shown here seems to be cell type-dependent, as Cav3.1 showed a uniform distribution in relay neurons of the thalamic dorsal lateral geniculate nucleus (Parajuli et al., 2010). Non-uniform distribution patterns has been previously described for different ion channels, including Cav2.3, GIRK and SK channels (Fernández-Alacid et al., 2011; Ballesteros-Merino et al., 2012, 2014; Parajuli et al., 2012).

Our data also show that the quantitative distribution of Cav3.1 and Cav3.2 differs in functionally distinct synapses and inputs. For instance, CA1 pyramidal cells receive glutamatergic inputs from several sources, all of them with different functional roles (Spruston and McBain, 2007). Interestingly, the high density of Cav3.2 in the *so* and *sr*, but low density in the *slm*, suggests its possible functional association with the inputs originating from other pyramidal cells, septal fibers and commissural fibers, as well as from the CA3 pyramidal cells, but not with the perforant pathway. Similarly, the very low density of Cav3.2 in the soma of pyramidal cells indicates a lack of association with the inhibitory synapses established by the basket cells. Understanding how the function of PCs is affected by this non-uniform distribution of Cav3.1 and Cav3.2 subunits will require detailed electrophysiological studies, but it suggests they could be involved in synaptic plasticity (Hildebrand et al., 2009).

Dendritic spines contained the highest densities of Cav3.1 and Cav3.2 immunoparticles, indicating their possible role at excitatory synapses. However, Cav3.1 was more concentrated than Cav3.2 at the edge of the excitatory synapse. The molecular mechanisms involved in the differential subcellular distribution of Cav3.1 and Cav3.2 subunits along the neuronal surface described here are still mostly unknown. One possible involved factor is the association with other proteins including G-protein-coupled receptors, G proteins, ion channels or enzymes (Chemin et al., 2006; Iftinca and Zamponi, 2009). Consistent with this idea, the distribution pattern of Cav3.1 in PC spines is strikingly similar to that of mGlu1α (López-Bendito et al., 2001), in agreement with their functional coupling, which contributes to the induction of plasticity at parallel fiber synapses (Hildebrand et al., 2009). Similarly, the distribution pattern of Cav3.2 in the spines of PCs and CA1 pyramidal cells, as well as along the active zone of axon terminals, is reminiscent of the distribution of the GABA_B_ receptor (Kulik et al., 2003; Luján and Shigemoto, 2006).

## Author Contributions

RL designed the project; CA, SG-M and MG-M performed histoblot analysis; RL and CA performed pre-embedding immunoelectron microscopy; RL and CA analyzed data; RL wrote the article.

## Conflict of Interest Statement

The authors declare that the research was conducted in the absence of any commercial or financial relationships that could be construed as a potential conflict of interest.
